# Low complication rate after same-day total hip arthroplasty: a retrospective, single-center cohort study in 116 procedures

**DOI:** 10.1080/17453674.2019.1637631

**Published:** 2019-07-05

**Authors:** Merete N Madsen, Maria L Kirkegaard, Malene Laursen, Jens R Larsen, Merete F Pedersen, Birgitte Skovgaard, Thomas PrynØ, Lone R Mikkelsen

**Affiliations:** Elective Surgery Centre, Silkeborg Regional Hospital, Denmark

## Abstract

Background and purpose — Length of hospital stay (LOS) following total hip arthroplasty (THA) has been markedly reduced. Recently, same-day THA (SD-THA) was introduced, and previous studies have indicated satisfactory safety. However, studies are heterogeneous and only a few report results on SD-THA when using a posterolateral surgical approach. Thus, our aim was to evaluate the feasibility of and complications after SD-THA when using a posterolateral approach.

Patients and methods — Consecutive patients scheduled for SD-THA between October 2015 and June 2016 were included. Eligibility criteria for SD-THA were: primary THA, motivation for same-day procedure, age > 18 years, ASA I or II, and the presence of a support person who could remain with the patient for 24 hours after surgery. A posterolateral surgical approach was used. Data were collected retrospectively from hospital records and the Danish National Patient Registry. Outcome measures were: complications during admission, LOS, causes of prolonged admission, and prevalence and causes of readmission at 90 days’ follow-up.

Results — 102 of 116 (88%) patients scheduled for SD-THA were discharged on the day of surgery. The remaining 14 patients were discharged the following day. Primary causes of prolonged admission were: dizziness/nausea, pain, and wound seepage. 7 patients had an estimated blood loss above 400 mL, but all were discharged as planned. No major complications occurred during admission. At follow-up, 3 patients had been readmitted due to pneumonia, wound infection, and dislocation, respectively.

Interpretation — The results indicate that SD-THA performed with a posterolateral approach is feasible and can be performed with a low complication rate in a selected group of patients.

Fast-track regimens have been shown to markedly reduce length of hospital stay (LOS) after total hip arthroplasty (THA) (Husted et al. 2012) without compromising morbidity and mortality (Husted 2012, Glassou et al. 2014). In recent years, patient discharge on the day of surgery (SD-THA) has been introduced, and newly published systematic reviews have concluded that outpatient arthroplasty (THA as well as TKA) may be a safe procedure in selected patients (Pollock et al. 2016, Kort et al. 2017, Hoffmann et al. 2018, Lovett-Carter et al. 2018). However, Pollock et al. (2016) also stated, that the majority of studies in the review were of poor quality. Additional studies, not included in the systematic reviews, support the findings of feasibility of SD-THA in selected patient groups (Parcells et al. 2016, Larsen et al. 2017, Klein et al. 2017, Berend et al. 2018, Kim et al. 2018, Toy et al. 2018) and to a lesser degree in unselected patients (Gromov et al. 2017). In addition, studies have reported satisfactory results regarding safety (Parcells et al. 2016, Basques et al. 2017, Courtney et al. 2017, Klein 2017, Larsen et al. 2017, Nelson et al. 2017, Berend 2018, Toy et al. 2018). Conversely, higher rates of postoperative complications, primarily medical complications, have been reported in 2 database studies comparing non-randomized groups of SD-THA patients and THA inpatients (Lovecchio et al. 2016, Otero et al. 2016).

Despite the already mentioned promising results, further research is needed. Available studies are heterogenous, i.e., due to differences in health care set-up, patient selection criteria, surgical approach, and duration of follow-up. Studies reporting on the posterolateral surgical approach are few (Gromov et al. 2017, Larsen et al. 2017, Springer et al. 2017) and only with a short follow-up period of 6 weeks or less. Thus, we evaluated feasibility of and complications after SD-THA when using a posterolateral approach and with 90 days’ follow-up.

## Patients and methods

### Design

The study was a retrospective cohort study conducted at a public hospital with free access and with patients being treated free of charge.

### Participants

All patients scheduled for SD-THA between October 2015 and June 2016 were included in the study. Eligibility criteria were: primary THA, motivation for and acceptance of a same-day procedure, age above 18 years, and a health condition categorized as ASA class I or II. In addition, an adult support person, who was capable of providing emotional and physical support to the patient during the first 24 hours after surgery, had to be available. The clinical pathway is presented in Table 1, see Supplementary data.

**Table 1.** Clinical pathway of THA patients with scheduled discharge on the day of surgery (SD-THA)

When What	Content
**Before admission**	
Clinical examination	A clinical examination was performed at the outpatient clinic.
	Eligibility for THA surgery and SD-THA was determined in a shared decision-process between the surgeon, the patient and, if possible, the support person.
Preoperative educational class	All patients and support persons were advised to attend a preoperative education class.
	An interdisciplinary team of health professionals explained the perioperative pathway, i.e., procedures of anesthesia, surgery, rehabilitation, postoperative movement restrictions, and expected postoperative pain and management.
**During admission**	
Surgery	SD-THA patients were operated as first or second case on the day in the operating room.
	Surgery was performed by three different surgeons, all using a standard posterolateral approach.
	The approach was not piriformis sparing but quad femoris was spared when possible. The prosthesis used was either uncemented or hybrid depending on the surgeon’s choice. An absorbent dry wound bandage was applied. No drains or urinary catheters were used
Anesthetic regimen	Standard noninvasive monitoring was established.
	Sedation was made available.
	Spinal injection of 10 mg bupivacaine (2 ml 0.5% Marcaine)
	Local anesthetic was not used.
Prescribed medicine	Given on the day of surgery only:
	Cefuroxime (IV 1,500 x 3) (the first dose is given prior to surgery)
	Xarelto (10 mg x 1) (given in the evening)
	Dexamethasone (8 mg IV x 1, given just before surgery) ^a^
	Standard package for pain control—on both the day of surgery and the following days:
	Paracetamol (1 g x 4) (first dose is given prior to surgery)
	Ibuprofen (400 mg x 3) (prescribed for 10 days, first dose is given prior to surgery)
	Pantoprazole (40 mg x 1) (prescribed for 10 days, first dose is given prior to surgery)
	Postoperative pain was managed per need with oxycodone (5–10 mg – max. x 6) based on repetitive NRS pain measurements. Though not being a specific discharge criterion, the aim was that the patients’ pain at rest and activity, respectively, should not exceed NRS 3 and 5.
Mobilization and radiographs	Full weight-bearing was allowed, and patients were mobilized as soon as ready (depending on the patient’s pain, well-being, and ability to control the leg). Postoperative radiographs were taken before discharge and were approved either on the day of surgery or the following day at the latest.
Discharge	If ready, patients were discharged according to plan before 9 p.m. on the day of surgery.
	Discharge criteria were the same as for standard fast-track THA patients: no wound seepage beyond expected levels, spontaneous urination, independent in showering and dressing, patient perceived to have sufficient pain control, patient generally feeling well, instructed by a physiotherapist in home-based exercises, capable of walking safely with a stick, capable of climbing stairs and knowledge of the movement restrictions.
**After discharge**	
Phone contact	The day after discharge, a nurse contacted the patients by telephone to check their physical and mental well-being.
Postoperative clinical control	3–4 weeks after surgery the patients were offered an individual clinical control by a physiotherapist at the hospital. This clinical visit finished the THA course at the hospital unless further controls were considered needed.

a The use of dexamethasone was gradually implied in the regime during fall 2015.

### Data collection

Data were retrospectively retrieved from hospital records and the Danish National Patient Registry (DNPR) (Lynge et al. 2011). Data were collected from admission until 90 days after discharge. The total number of patients receiving a THA during the study period was retrieved from the Business Intelligence Portal. No further data on patients following a standard fast-track procedure were collected.

Outcome measures related to feasibility were discharge readiness (before 9 p.m.), LOS, and causes of prolonged admission. Causes of prolonged admission and readmission were extracted from clinical notes. Outcome measures related to complication rate were: complications during admission (major intraoperative blood loss [estimated > 400 mL], blood transfusion, fracture, hip dislocation, other), prevalence and causes of readmissions (readmission defined as a patient admitted to hospital ward and taking up a bed).

Furthermore, data were collected regarding patient demographics (sex, age, marital status, employment status [defined as employed, job seeking, pensioner/retired, sick leave]), ASA class, previous lower limb arthroplasty (THA/TKA), primary diagnosis, duration of surgery, type of prosthesis, use of IV glucocorticosteroids, and pain intensity during admission measured in both rest and activity using the Numeric Range Scale (NRS).

### Statistics

Data are presented using descriptive statistics. Categorical variables are described in numbers and proportions with 95% confidence interval (Cl). All continuous variables were non-normally distributed and therefore reported in median with either interquartile range (IQR) or range or both. Readmission rate was reported for the group of patients in total and furthermore described in numbers for the group of patients discharged before and after 9 p.m., respectively.

In an exploratory analysis, associations between patient characteristics (age, BMI, sex, ASA class) and prolonged admission were tested with the Wilcoxon rank sum test for continuous variables and by estimating relative risk with CI for categorical variables.

Data entry and data analysis was performed in Epidata 3.1 (https://www.epidata.dk/) and IBM SPSS Statistics 24 (IBM Corp, Armonk, NY, USA), respectively. Supplemental analysis was performed in Stata 15.

### Ethics, funding, and conflicts of interest

The study was performed in accordance with the Helsinki Declaration of 1975 (revised in 2013) and approved by the Danish Data Protection Agency (1-16-02-165-17) and the management at the Hospital. The Danish Health Data Board provided the requested data from the Danish National Patient Registry. According to Danish law, no further approvals were required. No grants were received. All authors declare no conflicts of interest.

## Results

During the study period, 669 primary THA procedures were performed and, of these, 116 (in 115 patients) were scheduled for SD-THA. Data were available for all patients at 90 days’ follow-up (Figure).

Baseline patient characteristics are given in Table 2 and surgery characteristics, use of dexamethasone, and postoperative pain intensity are presented in Table 3. The NRS pain score did not exceed 3 at rest among 58% of the patients and 5 during activity among 80%.

**Table 2.** Baseline patient characteristics. Values are frequency unless otherwise stated

Variable (n = 115 **^a^**, unless stated otherwise)	
Male sex	72
Age, median (range)	62 (29–83)
Married/spouse (n = 114)	107
BMI (n = 114), median (range)	27 (19–38)
Employment status (n = 111)	
employed	61
job seeking	5
pensioner/retired	44
sick leave	1
ASA class (I/II)	59/56
Previous THA or TKA	24
Primary diagnosis (n = 114)	
hip arthrosis	104
rheumatoid arthritis	2
fracture sequelae	2
other	6

a The number is 115, as 1 patient had 2 procedures performed during the study period. Only data from this patient’s first operation are included.

**Table 3.** Surgery characteristics, use of dexamethasone and postoperative pain intensity

Factor (n = 116, unless stated otherwise)	
Duration of surgery (n = 115), min.	
median (IQR)	32 (27–41)
Type of prosthesis, n	
uncemented/hybrid	114/2
Estimated blood loss (n = 92), mL	
median (IQR)	150 (100–225)
Use of steroid (dexamethasone), n	
yes/no	97/19
Pain (NRS **^a^**), median (IQR) [range]	
rest^b^	1.5 (0.1–2) [0–5]
activity (n = 90)^c^	3.0 (2–4) [0–6]

a Numeric Range Scale.

b NRS pain at rest was measured between 1 and 6 times after surgery.

c NRS pain during activity was measured between 0 and 4 times after surgery.

### Feasibility

From 669 elective primary THA procedures, 116 (17%) were scheduled for SD-THA. In 102 of 116 procedures (88%), patients were discharged as planned. Median LOS was 10.5 hours (range: 6–12 hours) from end of surgery until discharge. The 14 patients with prolonged admission had a median LOS of 25 hours and all were discharged the day after surgery (Table 4).

**Table 4.** Reasons for prolonged admission

Reason (n = 14)	n
Dizziness and nausea	5
Pain	3
Wound seepage	4
Personal matters	
spouse concerned	1
patient concerned about discharge	1

In an exploratory univariate analysis, it was found that female sex (RR 3.1 [CI 1.1–8.5]) and ASA class II compared with ASA class I (RR 3.8 [CI 1.1–13]) were significantly associated with higher risk of prolonged admission. Neither age (p = 0.9) nor BMI (p = 0.8) was found to be statistically significantly associated with prolonged admission.

### Complications

Major bleeding occurred in 7 of 116 patients but all were discharged on the day of surgery as planned. Estimated blood losses in these 7 patients were between 450 mL and 1000 mL. There was no statistically significant difference (p = 0.5) between estimated blood loss among the patients with prolonged admission (median: 200 mL) compared with the patients discharged as planned (median: 150 mL). No hip dislocations, fractures, blood transfusions, or any other major complications occurred during admission.

At 90 days’ follow-up 3 patients had been readmitted. The readmissions occurred 3, 14, and 17 days after surgery, respectively, and were due to pneumonia (1), hip dislocation (1), and wound infection (1). 2 of the patients readmitted had been discharged on day of surgery, while the patient readmitted due to dislocation was discharged the day after surgery due to wound seepage.

## Discussion

Our primary findings were a high proportion (88%) of patients being discharged on the day of surgery as planned, no occurrence of major complications and a 2.6% readmission rate, indicating that SD-THA in this selected group of patients was feasible and safe.

### Feasibility

In this study, 17% of all primary THA patients were eligible for and accepted SD-THA. Of these, 88% were successfully discharged on the day of surgery. Altogether, 102/669 (15%) from our total group of THA patients were discharged on the day of surgery. This result is in accordance with the findings of Gromov et al. (2017), who reported that in an unselected group of THA patients 15% could be discharged on the day of surgery. Even though it is reported to be conducted in unselected patients, nevertheless, Gromov et al. described that to be eligible for same-day surgery patients should be classified as ASA class < 3, operated as number 1 or 2 in the operating room, and have an adult person present at home for at least 24 hours after discharge. These criteria are similar to part of our patient selection criteria, thus making our results likely to be comparable.

The number of primary THA patients discharged on the day of surgery from our hospital could possibly be higher than the reported 102 patients, as some patients might have been ready for discharge even though this was not planned in advance. This was the case in the randomized controlled trial by Goyal et al. (2017), in which 18 of 108 patients allocated to be inpatients (discharge the day after surgery) fulfilled the criteria for discharge and chose to leave hospital on the day of surgery. Furthermore, whether all 669 primary THA patients in our study were actually screened for eligibility to be offered a same-day procedure was not registered. Thus, there may be a potential to increase the proportion of SD-THA patients. On the other hand, neither was it registered how many patients were offered but declined to be scheduled for SD-THA.

We consider the result of 88% patients being discharged according to plan to be satisfactory, as it is comparable to the result of 85% from our pilot study (Larsen et al. 2017). Comparing our result with studies performed in a traditional hospital setting, only 2 more studies report the use of a posterolateral surgical approach similar to our procedure (Gromov et al. 2017, Springer et al. 2017). Springer et al. do not report the proportion of patients scheduled for SD-THA discharged as planned, while, as mentioned earlier, the result from Gromov et al. is in accordance with our result. In studies using other surgical approaches, the proportion of patients scheduled for SD-THA and discharged as planned varied between 76% and 100% (Berger et al. 2009, Dorr et al. 2010, Aynardi et al. 2014, den Hartog et al. 2015, Goyal et al. 2017). Furthermore, 4 studies, performed in ambulatory surgical centers, had 94% or more discharged on the day of surgery (Parcells et al. 2016, Klein et al. 2017, Berend et al. 2018, Toy et al. 2018), but the different set-up should be taken into account when comparing our result with these studies.

### Complications during admission

Postoperative level of hemoglobin was not routinely tested but required per need based on the patient’s well-being and blood loss. A postoperative hemoglobin level lower than 4.3 mmol/L (69 g/L) and/or clinical signs of anemia (not responding on fluid therapy) would constitute transfusion requirement in non-ischemic heart disease patients (Danish Health and Medicines Authority 2015), but in this study none of the patients scheduled for SD-THA required a blood transfusion. This is less than in the total group of THA patients at our hospital, as the proportion of THA patients requiring a blood transfusion within 7 days after surgery was 3.0% in 2016 and 2.3% in 2015 (DHR Annual Report 2017). Our result supports the findings from an American database study, stating that blood transfusion was less for SD-THA than for THA inpatients (Nelson et al. 2017). However, other studies based on the same database have found no difference in transfusion rate (Lovecchio et al. 2016, Basques et al. 2017) or even a higher rate (Otero et al. 2016). Although comparison should be made with caution, we find our result satisfactory.

7 patients in our study had an estimated blood loss above 400 mL, but they were all feeling well (thus, no clinical signs of anemia) and were ready for discharge on the day of surgery. In 24 patients, the estimated blood loss was not registered. 5 of these patients were not discharged on the day of surgery but none of them was readmitted during follow-up. In summary, our results indicate that a blood loss above 400 mL did not have an essential influence on the patient’s discharge readiness. However, due to missing data, this finding should be considered with caution. Introduced as a safety consideration, one of the criteria for discharge by Gromov et al. (2017) was an estimated blood loss < 500 ml. That criterion was not fulfilled in 27% of their patients, whereas in our study the corresponding number was only 5%, thus indicating a well-functioning blood-saving strategy in our set-up.

We found no other major complications during admission. This result is comparable to our previous results from 2015, in which only one minor perioperative complication occurred (drilling fracture during surgery) (Larsen et al. 2017). Our results thereby support the findings in previous studies reporting few or no acute major complications (Berger et al. 2009, Aynardi et al. 2014, den Hartog et al. 2015, Klein et al. 2017, Springer et al. 2017, Berend et al. 2018, Toy et al. 2018).

14 patients had prolonged admission, among whom 12 were due to dizziness and nausea, pain, and wound seepage, with none of the causes being dominating. These causes are well known from other studies (Berger et al. 2009, Dorr et al. 2010, den Hartog et al. 2015, Goyal et al. 2017, Gromov et al. 2017, Springer et al. 2017, Berend et al. 2018, Kim et al. 2018). It should be considered that dexamethasone, which is supposed to reduce pain and nausea, was gradually employed as a standard procedure in late 2015. Thereby, patients operated at the beginning of the study period were less likely to have received it. Though not statistically significant, the proportion of patients with prolonged admission was higher among patients who did not receive dexamethasone (21%) compared with those who did (10%). Thus, it could be hypothesized that, given all eligible patients had received dexamethasone, the number of patients discharged as planned could have been higher.

### Readmissions

Due to low complication rates, it requires a vast number of patients to truly compare complication rates after SD-THA with a population of standard fast-track THA. As an approximative alternative, we find it relevant to evaluate the readmission rate in our study with readmission rates reported in the Danish Hip Arthroplasty Register in 2016 (DHR Annual Report 2017). Although it is a problem to compare with populations, which both comprises part of our study population and who furthermore in general are older and less healthy than in the current study, it has some advantages. The register uses the same definition of readmission as in the current study and a 30-day follow-up, which is comparable to the current study, as all our readmissions occurred within the first 30 postoperative days. Our raw readmission rate (2.6%) did not exceed the raw estimates for the total primary THA populations, which were 5.9% in our local hospital setting and 9.7% in total in Denmark, respectively (DHR Annual Report 2017). The result is in accordance with comparative studies, in which similar readmission rates between SD-THA and THA inpatients were found (Lovecchio et al. 2016, Basques et al. 2017, Courtney et al. 2017, Goyal et al. 2017, Springer et al. 2017). Also, our result is similar to that of other observational studies (Berger et al. 2009, den Hartog et al. 2015, Dorr et al. 2010, Larsen et al. 2017, Toy et al. 2018) reporting between 0% (Larsen et al. 2017, Dorr et al. 2010) and 3.7% (den Hartog et al. 2015).

### Study limitations and strengths

The main limitation concerns selection in patient material. Our patients differed from the total THA population in terms of being younger, healthier, and with a higher proportion of male sex. Furthermore, no data were available on whether all eligible patients were offered SD-THA, or the reasons for patients not being scheduled for SD-THA. Thus, beside the predefined criteria, there is a potential risk of our patient population being further selected. For instance, cognitive status, personal resources, and distance from the patient’s home to the hospital are not part of the eligibility criteria, but may be accounted for when deciding whether to be scheduled for SD-THA. These factors could influence both the surgeon’s decision to offer SD-THA and also the patient’s willingness and motivation to accept the offer. However, as our result is in accordance with the result from Gromov et al. (2017), the risk of further selection might be minor. In addition, extension of the result to different settings as well as in other countries may be of limited value. Due to the retrospective design, we had areas with missing data (i.e., estimated blood loss), and other variables with data not being systematically registered (i.e., pain). Additionally, causes of prolonged admission were based on clinical notes and not registered in predefined categories, thus potentially increasing the risk of misclassification. However, as data were registered independently of research, the risk of differential misclassification is considered low. Furthermore, after discharge, minor complications not leading to readmission were not registered. This should be considered when comparing with other studies. Primarily, the strength of the study was the 100% follow-up regarding readmissions. Secondly, data reflect daily practice, which increases the validity of clinical effectiveness.

In summary, 88% of patients scheduled for SD-THA, performed with a posterolateral approach, were discharged on the day of surgery. Primary causes of prolonged admission were dizziness/nausea, pain, and wound seepage. 6% of the patients had an estimated blood loss above 400 mL, but all were discharged as planned without any blood transfusions. No major complications occurred during admission. At 90 days’ follow-up, 3 patients had been readmitted due to pneumonia or wound infection or hip dislocation, respectively. Thus, we conclude that SD-THA is feasible and can be performed with a low complication rate in a selected group of patients.

### Supplementary data

Table 1 is available as supplementary data in the online version of this article, http://dx.doi.org/10.1080/17453674.2019.1637631

**Figure:**
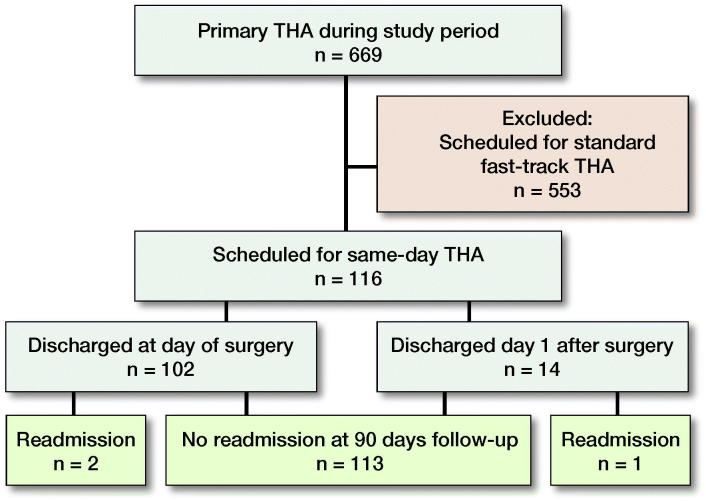
Participant flow.

## Supplementary Material

Supplemental Material
