# Supplementary material for: Low complication rate after same-day total hip arthroplasty: a retrospective, single-center cohort study in 116 procedures
Source: Acta Orthop. 2019 Jul 5;90(5):439–44. doi: 10.1080/17453674.2019.1637631 (PMC6746288; doi:10.1080/17453674.2019.1637631)
Supplement: Supplemental Material [file IORT_A_1637631_SM5945.pdf]

Supplementary data

Table 1. Clinical pathway of THA patients with scheduled discharge on the day of surgery (SD-THA)

When	What	Content
**Before admission**		
	Clinical examination	A clinical examination was performed at the outpatient clinic.<br>Eligibility for THA surgery and SD-THA was determined in a shared decision-process between the surgeon, the patient and, if possible, the support person.
	Preoperative educational class	All patients and support persons were advised to attend a preoperative education class.<br>An interdisciplinary team of health professionals explained the perioperative pathway, i.e., procedures of anesthesia, surgery, rehabilitation, postoperative movement restrictions, and expected postoperative pain and management.
**During admission**		
	Surgery	SD-THA patients were operated as first or second case on the day in the operating room.<br>Surgery was performed by three different surgeons, all using a standard posterolateral approach.<br>The approach was not piriformis sparing but quad femoris was spared when possible. The prosthesis used was either uncemented or hybrid depending on the surgeon's choice. An absorbent dry wound bandage was applied. No drains or urinary catheters were used
	Anesthetic regimen	Standard noninvasive monitoring was established.<br>Sedation was made available.<br>Spinal injection of 10 mg bupivacaine (2 ml 0.5% Marcaine)<br>Local anesthetic was not used.
	Prescribed medicine	Given on the day of surgery only:<br>Cefuroxime (IV 1,500 x 3) (the first dose is given prior to surgery)<br>Xarelto (10 mg x 1) (given in the evening)<br>Dexamethasone (8 mg IV x 1, given just before surgery) [a]<br>Standard package for pain control—on both the day of surgery and the following days:<br>Paracetamol (1 g x 4) (first dose is given prior to surgery)<br>Ibuprofen (400 mg x 3) (prescribed for 10 days, first dose is given prior to surgery)<br>Pantoprazole (40 mg x 1) (prescribed for 10 days, first dose is given prior to surgery)<br>Postoperative pain was managed per need with oxycodone (5–10 mg – max. x 6) based on repetitive NRS pain measurements. Though not being a specific discharge criterion, the aim was that the patients' pain at rest and activity, respectively, should not exceed NRS 3 and 5.
	Mobilization and radiographs	Full weight-bearing was allowed, and patients were mobilized as soon as ready (depending on the patient's pain, well-being, and ability to control the leg). Postoperative radiographs were taken before discharge and were approved either on the day of surgery or the following day at the latest.
	Discharge	If ready, patients were discharged according to plan before 9 p.m. on the day of surgery.<br>Discharge criteria were the same as for standard fast-track THA patients: no wound seepage beyond expected levels, spontaneous urination, independent in showering and dressing, patient perceived to have sufficient pain control, patient generally feeling well, instructed by a physiotherapist in home-based exercises, capable of walking safely with a stick, capable of climbing stairs and knowledge of the movement restrictions.
**After discharge**		
	Phone contact	The day after discharge, a nurse contacted the patients by telephone to check their physical and mental well-being.
	Postoperative clinical control	3–4 weeks after surgery the patients were offered an individual clinical control by a physiotherapist at the hospital. This clinical visit finished the THA course at the hospital unless further controls were considered needed.

[a] The use of dexamethasone was gradually implied in the regime during fall 2015.
